# WebHERV: A Web Server for the Computational Investigation of Gene Expression Associated With Endogenous Retrovirus-Like Sequences

**DOI:** 10.3389/fmicb.2018.02384

**Published:** 2018-11-05

**Authors:** Konstantin Kruse, Martin Nettling, Nadine Wappler, Alexander Emmer, Malte Kornhuber, Martin S. Staege, Ivo Grosse

**Affiliations:** ^1^Institute of Computer Science, Martin Luther University Halle-Wittenberg, Halle, Germany; ^2^Department of Surgical and Conservative Pediatrics and Adolescent Medicine, Martin Luther University Halle-Wittenberg, Halle, Germany; ^3^Department of Neurology, Martin Luther University Halle-Wittenberg, Halle, Germany; ^4^Department of Neurology, Helios Hospital, Sangerhausen, Germany; ^5^German Centre for Integrative Biodiversity Research (iDiv) Halle-Jena-Leipzig, Leipzig, Germany

**Keywords:** endogenous retroviruses, HERVs, Hodgkin lymphoma, CYP4Z1, BLAST, DRUMS, database, web server

## Abstract

More than eight percent of the human genome consists of human endogenous retroviruses (HERVs). Typically, the expression of HERVs is repressed, but varying activities of HERVs have been observed in diseases ranging from cancer to neuro-degeneration. Such activities can include the transcription of HERV-derived open reading frames, which can be translated into proteins. However, as a consequence of mutations that disrupt open reading frames, most HERV-like sequences have lost their protein-coding capacity. Nevertheless, these loci can still influence the expression of adjacent genes and, hence, mediate biological effects. Here, we present WebHERV (http://calypso.informatik.uni-halle.de/WebHERV/), a web server that enables the computational prediction of active HERV-like sequences in the human genome based on a comparison of genome coordinates of expressed sequences uploaded by the user and genome coordinates of HERV-like sequences stored in the specialized key-value store DRUMS. Using WebHERV, we predicted putative candidates of active HERV-like sequences in Hodgkin lymphoma (HL) cell lines, validated one of them by a modified SMART (switching mechanism at 5′ end of RNA template) technique, and identified a new alternative transcription start site for cytochrome P450, family 4, subfamily Z, polypeptide 1 (CYP4Z1).

## 1. Introduction

Human endogenous retroviruses (HERVs) are an increasingly recognized part of the human genome that entered the germline in the course of evolution and today comprise more than eight percent of it (Makałowski, 2001; de Parseval and Heidmann, 2005). Most of these sequences are no longer protein coding due to mutations, but there are several exceptions such as syncytins, which exert a physiological function in placenta development (Denner, 2016).

HERV envelope sequences might be translated and exert immuno-regulatory activity (Kassiotis and Stoye, 2016), and it is a matter of current research to which degree the high number of HERV-like sequences without intact open reading frames have physiological or patho-physiological functions (Moyes et al., 2007; Ruprecht et al., 2008; Voisset et al., 2008; Dolei and Perron, 2009). Interestingly, HERV-like sequences can act as regulatory elements for adjacent genes, and the promoter activity of such elements has been demonstrated to influence the activity of oncogenes in lymphoma cells (Lamprecht et al., 2010).

Cloning strategies for the identification of expressed HERV loci have been developed (Wilkinson et al., 1993), but the experimental effort of these methods is high. In contrast, the experimental effort of next-generation-sequencing-based approaches is low, but the challenge of these approaches is that analyzing the resulting data in a coherent manner requires some non-negligible bioinformatics effort.

Here, we describe the web server WebHERV that enables genome-wide analyses of the proximity of differentially expressed genes and HERV-like sequences. These analyses are based on sets of genome coordinates representing transcriptionally active gene loci generated for example by micro-array or RNA-seq experiments and sets of coordinates of HERV-like sequences retrieved from an integrated DRUMS database (Nettling et al., 2014).

Co-expression of HERV-like sequences and host genes may lead to differential transcript abundances of these genes in the vicinity of HERV-like sequences. In turn, the observation of such expression patterns might be indicative for the activity and for the potentially physiological or patho-physiological functions of the associated HERV-like sequences.

The current implementation of WebHERV is based on a database of sequences with high similarities to HERV loci as defined by Villesen et al. (2004) without any additional size limitation and without further sequence restriction. In addition, WebHERV provides the option of using RepeatMasker coordinates as alternative source for HERV-like sequences.

The rest of the paper is structured as follows. In section 2, we present the underlying data of WebHERV, the architecture of WebHERV, and one exemplary biological application. In section 3, we use WebHERV for predicting putative candidates of HERV-like sequences that might influence the transcription of genes in Hodgkin lymphoma (HL) cell lines.

## 2. Materials and methods

In this section, we first describe the prediction of HERV-like sequences in the human genome. Second, we describe the data storage. Third, we describe the functionality of the web server and the data flow.

### 2.1. Predicting HERV-like sequences

We used the predicted HERVs published by Villesen et al. (2004) as basis to search for the complete set of HERV-like sequences in two versions of the human reference genome using BLAST version blast-2.6.0-linux (Altschul et al., 1990) with an *E*-value threshold of 10^−10^. This BLAST search identified more than 4 × 10^8^ HERV-like sequences in both of the human reference genome versions hg18 and hg19, which we integrated into the DRUMS database at the back-end of WebHERV.

The sequences from Villesen et al. (2004) preferentially include long and almost complete HERV-like sequences with partially intact open reading frames. We accepted this bias because the expression of such sequences might be of particular interest in the context of human diseases.

However, this bias might not be desirable or sometimes not be acceptable for other applications. Hence, we developed WebHERV in such a way that the user can easily extend or modify the database and include for example other HERV-like sequences with lower sequence similarity to typical retroviruses or other repetitive elements.

In order to allow the identification of long terminal repeats (LTRs) and other elements with lower sequence similarities to retroviruses, we also integrated the RepeatMasker coordinates of LTR elements into the DRUMS database of WebHERV.

### 2.2. HERV loci store

Traditional relational databases like MySQL have severe problems with the integration of—and with performing queries against—more than 10^8^ sequences (Nettling et al., 2014). Hence, we used the tailored storage system DRUMS (Nettling et al., 2014) as HERV loci store at the back-end of WebHERV, which allows both a smooth integration and smooth queries of more than 4 × 10^8^ HERV-like sequences.

### 2.3. WebHERV

The front-end of WebHERV is a web application based on JavaServerFaces and publicly available at http://calypso.informatik.uni-halle.de/WebHERV/. The user can upload a file with genomic positions derived from arbitrary sources of RNA-seq or micro-array data. Alternatively, the user can upload a file with probe set IDs of the Affymetrix Human Exon 1.0 ST array platform, as WebHERV stores the positional information of these probe sets in an additional SQLite database.

The user can specify several search parameters, which are described in detail next to the corresponding input field. In addition, a step-by-step user instruction is available for downloaded at http://calypso.informatik.uni-halle.de/WebHERV/resources/docs/InstructionsWebHERV.pdf. Finally, pressing the “submit” button starts the search for HERV-like sequences in the HERV loci store.

**Figure 1:**
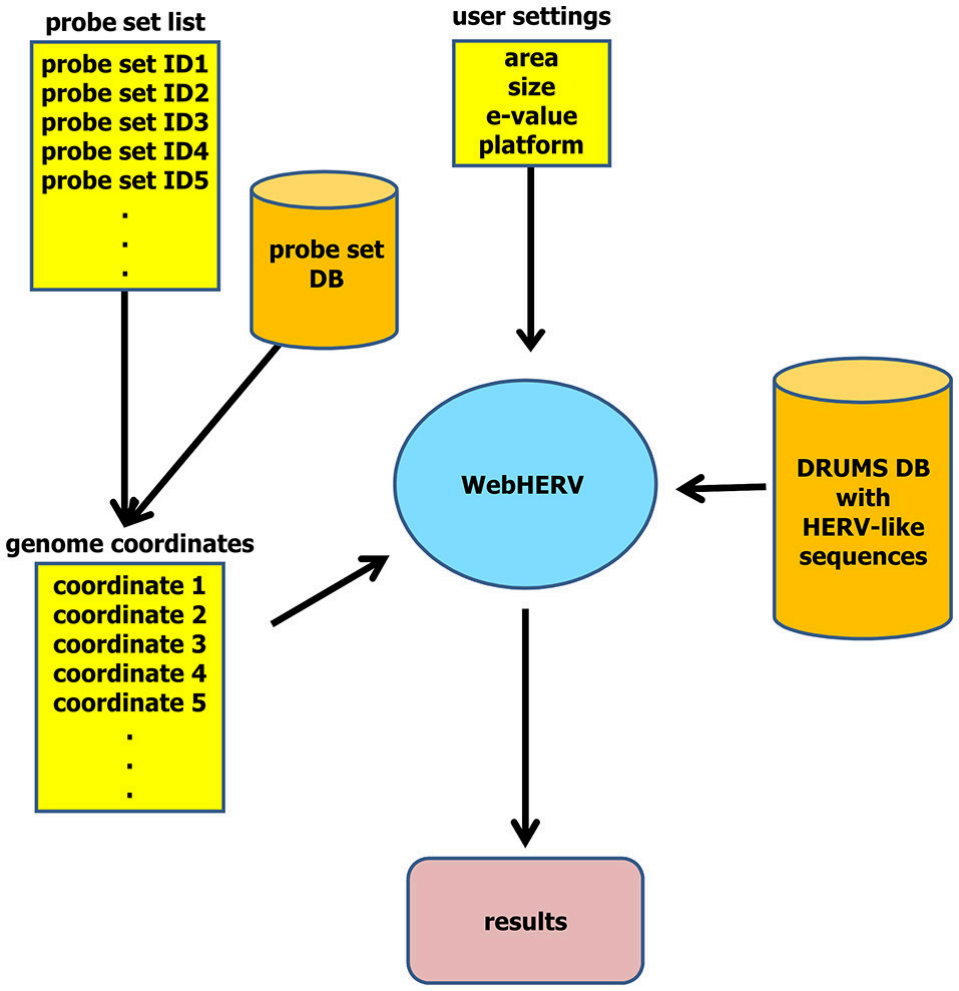
WebHERV data flow. The user can upload a file with genome coordinates or a file with probe set IDs that are then being transformed into genome coordinates utilizing the integrated probe set database. The user can then set search parameters and start the search of HERV-like sequences in the chromosomal neighborhood of these genome coordinates.

The results are interactively represented on a separate page. For each genomic position—or alternatively for each probe set—WebHERV displays all HERV-like sequences and their *E*-values found by the specified search parameters and makes these data available for download as CSV-file. The complete data flow is illustrated in Figure 1.

### 2.4. Biological application

In this section, we describe one application of WebHERV to the analysis of HL data. All analyzed data are available from the Gene Expression Omnibus (GEO) database. RNA from HL cell lines was isolated using TRIzol (Invitrogen, Karlsruhe, Germany) following the manufacturer's protocol.

Micro-array data (Affymetrix Human Exon 1.0 ST arrays) from HL cell lines were generated as described (Kewitz and Staege, 2013). For comparison, micro-array data from normal B cells (Nikitin et al., 2010) and normal blood cells (Shehadeh et al., 2010) were used. All Affymetrix cel files (GSE18838 for normal blood cells, GSE20200 for normal B cells, and GSE47686 for HL cell lines) were downloaded from the GEO database and processed using the Affymetrix Expression Console software (build 1.4.1.46; annotation file version huex-1-0stv2.na36.hg19).

From the mentioned data sets GSE18838 and GSE20200 only normal blood cells (11 samples) or normal B cells (4 samples) were used. Additional cel files (Affymetrix HG-U133Plus2.0 arrays) from HL samples (GSE12453, GSE12427, GSE20011, GSE25986, GSE39134, GSE7303, and GSE12453) and a panel of normal tissues from the human body atlas GSE7307 were also downloaded from the GEO database (Roth et al., 2006; Brune et al., 2008; Liu et al., 2010; Köchert et al., 2011; Steidl et al., 2011, 2012). Probe sets were pre-filtered by using MAFilter (Winkler et al., 2012) on the basis of a simple fold change calculation (Witten and Tibshirani, 2007; Draghici, 2011).

Probe sets were considered to be differentially expressed if the median signal intensity in one group (HL cell lines or normal blood cells) exceeded the 75th percentile of the signal intensities in the other group at least 10 times. Dividing the 50th percentile of one group by the 75th percentile of the other group has the advantage that outliers have only a low impact on the ratios.

When performing reverse transcription-polymerase chain reactions (RT-PCRs), two micrograms of RNA were transcribed into cDNA using the qScriptTM cDNA SuperMix (Quantabio, Beverly, MA, USA) following the manufacturer's protocol. RT-PCR was performed using a total volume of 25 μl with final concentrations of 10 pM forward and 10 pM revers primer, 200 μM dNTP Mix (ThermoFischer Scientific, Waltham, MA, USA), 1x Go-Taq-Buffer (Promega, Fitchburg, WI, USA), 0.04 U/μl GoTaq DNA-Polymerase (Promega), and 2 μl cDNA.

The following RT-PCR procedure was used: (i) 94°C, 5 min; (ii) 94°C, 30 s; (iii) 60°C, 30 s; (iv) 72°C, 45 s; (v) 72°C, 5 min. Steps (i) to (iv) were repeated 30 times. The following primers were used: CYP4Z1-E1-3: 5′-ttc ttg ctg ctg atc ctc ct-3′, 5′-ccc agg att caa gga ttt tg-3′; CYP4Z1-HERVLE: 5′-tca gca aac tat cgc aag ga-3′, 5′-tag ggg ttg tgg tga aga gc-3′.

Transcripts of CYP4Z1 in HL cells were characterized by using a modified SMART (switching mechanism at 5′ end of RNA transcript) technique as described in Kewitz et al. (2014). Sequencing of RT-PCR products was performed using the BigDye Terminator V1.1 Cycle Sequencing Kit (Life Technologies, Austin, TX, USA). The sequences were analyzed by BLAST (Altschul et al., 1990), and splice-site prediction was performed by Human Splicing Finder (Desmet et al., 2009).

## 3. Results and discussion

We tested the functionality of the web server by using two random lists of 60,000 probe sets generated by the MySQL random function. The percentages of probe sets that were identified as being HERV associated increased monotonically with increasing distances or increasing *E*-values.

795 probe sets (1.33%) and 873 probe sets (1.46%) from the two random lists of probe sets were identified as being HERV associated, respectively, when using no size limitation, an *E*-value threshold of 10^−100^, and a distance of 0 base pairs. These percentages increased to 3,291 (5.50%) and 3,267 (5.46%) when using an *E*-value threshold of 10^−10^.

HL is a lympho-proliferative disease with known re-activation of HERV-like sequences (Lamprecht et al., 2010; Staege et al., 2014). The majority of HL patients can be cured today, but the toxicity of the used therapy regimes is high. Hence, major efforts are being spent worldwide to identify novel targets that allow the development of less toxic treatment strategies in the future.

HERV-like sequences associated with differential gene expression might possibly represent such targets. Hence, one of our main research topics is the characterization of HL, and so we analyzed micro-array data from HL cell lines (Kewitz and Staege, 2013) in comparison to normal blood cells (Shehadeh et al., 2010) using WebHERV. From the blood data cell set, we only used healthy control samples.

We filtered probe sets with the highest signal intensities in HL cells compared to blood cells and *vice versa* as described in section 2 and obtained 4,329 up-regulated and 4,994 down-regulated probe sets in HL cells in comparison to normal blood cells. 4,306 and 4,918 of these probe sets have genome coordinates, and we provide these two lists of probe sets as sample files on the WebHERV homepage.

**Figure 2:**
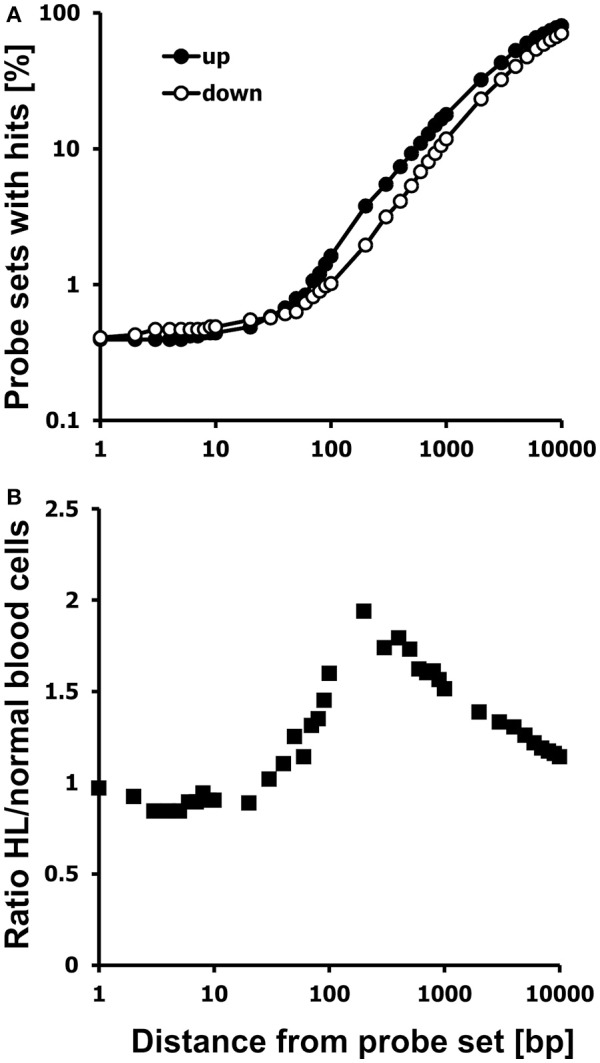
Probe sets with high signal intensities in HL cells are located preferentially in the neighborhood of HERV-like sequences. **(A)** Chromosomal neighborhoods of probe sets with high expression in HL cells (closed circles; GEO data set GSE47686) or normal blood cells (open circles; GEO data set GSE18838) were analyzed for the presence of HERV-like sequences. Presented are the percentages of probe sets with hits in the distance between 1 and 10,000 bp. **(B)** Presented is the ratio of the two percentages of probe sets with hits from up-regulated and down-regulated probe sets. The HERV association of HL specific probe sets is most pronounced at a distance of approximately 200 bp from the probe set.

We analyzed these probe sets for the presence of neighboring HERV-like sequences by WebHERV using the hg19 database and found that probe sets with higher signal intensities in HL cell lines were located more often in the vicinity of HERV-like sequences than probe sets with higher signal intensities in normal blood cells (Figure 2).

The percentage of probe sets with HERV-like sequences in the neighborhood increases in both cell types with increasing distances from the probe sets. However, a higher percentage of probe sets with high signal intensities in HL cells is located in the vicinity to HERV-like sequences.

We found 2,575 of the up-regulated probe sets (59.80%) and only 2,335 of the down-regulated probe sets (47.48%) to be associated with HERV-like sequences using a distance between 0 and 5,000 bp from the probe sets, an *E*-value threshold of 10^−100^, and no size restriction. In this data set, the HERV association was most pronounced at around 200 bp from the probe sets (Figure 2), but in other data sets the optimal distance might be different, so we set the default distance of WebHERV to 1,000 bp.

We found 163 from the up-regulated probe sets (3.79%) and 96 down-regulated probe sets (1.95%) in the neighborhood of HERV-like sequences using a distance of 200 bp. The percentage of HERV-associated probe sets reaches the limit of 100% and the ratio of HERV-associated probe sets in HL and normal cells reaches the limit of 1.0 with increasing distances, so we limited this analysis to a distance of 10 kb.

We found several genes among the HL specific probe sets associated with HERV-like sequences with a known high expression in HL (Staege et al., 2008, 2015; Hermes et al., 2016) such as the cancer/testis antigen PRAME (preferentially expressed antigen in melanoma), the cytokine EBI3 (Epstein-Barr virus induced 3), the chemokine fractalkine (CX3CL1), fascin (FSCN), or topoisomerase 2A (TOP2A). We also observed a high expression of all of these genes in HL cells in comparison to isolated B cells (Supplementary Figure 1).

**Figure 3:**
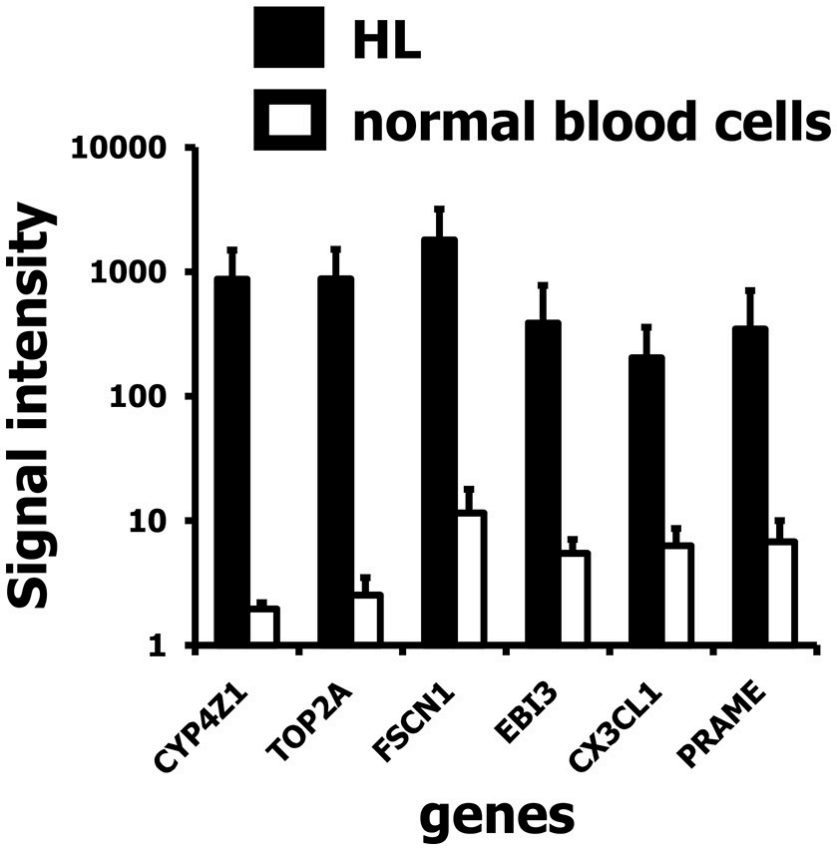
Signal intensities of six up-regulated genes in HL cell lines in the vicinity of HERV-like sequences. Presented are means and standard deviations for probe sets identified as associated with HERV-like sequences in HL cell lines (closed bars; GEO data set GSE47686) and normal blood cells (open bars; GEO data set GSE18838). A high up-regulation in HL was found for a probe set corresponding to the gene cytochrome P450, family 4, subfamily Z, polypeptide 1 (CYP4Z1).

**Figure 4:**
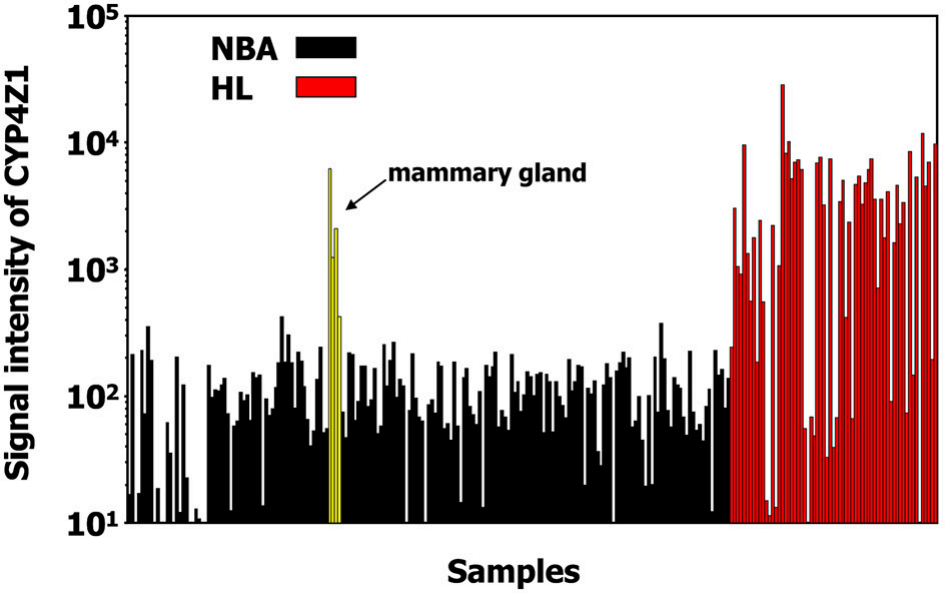
CYP4Z1 is an HL associated gene. Expression of CYP4Z1 in HL samples (from GEO data sets GSE12453, GSE12427, GSE20011, GSE25986, GSE39134) and normal tissues (from GEO data sets GSE7307) was assessed in micro-array data sets from the GEO database (Affymetrix HG-U133Plus2.0 micro-array data). High signal intensities were observed in the majority of HL samples. From the normal tissues only mammary gland expresses CYP4Z1.

In addition, we found a high up-regulation in HL cells for a locus on chromosome 1 corresponding to cytochrome P450, family 4, subfamily Z, polypeptide 1 (CYP4Z1; Figure 3). Independent micro-array data (Affymetrix HGU133Plus2.0 arrays) suggest that CYP4Z1 is indeed an HL associated gene. High signal intensities were observed in the majority of HL samples, and only mammary gland expressed CYP4Z1 in normal tissues (Figure 4).

We asked whether HERV-like sequences in the CYP4Z1 gene might influence the transcription of this gene or *vice versa*. A HERV-like sequence is located in the intron between exons 9 and 10 of the reference sequence, and we used a SMART (switching mechanism at 5′ end of RNA transcript) technique for the identification of the 5′ end of the CYP4Z1 transcripts.

**Figure 5:**
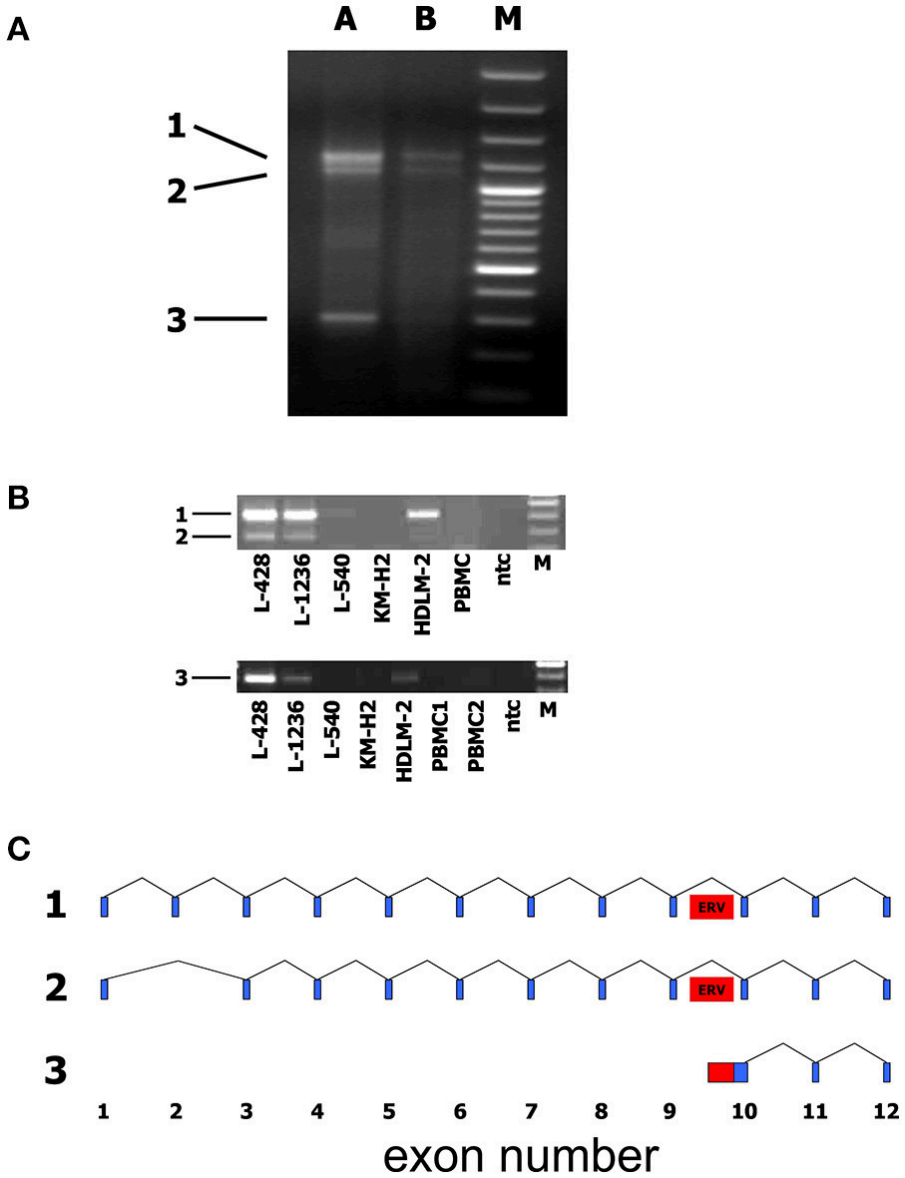
Identification of alternative transcripts and promoters of human CYP4Z1. **(A**) The SMART technique was used for the identification of the 5′ end of CYP4Z1 transcripts. Three different transcripts were identified. A + B: RNA from two different samples of the HL cell line L-428 was used for SMART; M: DNA size marker. **(B)** Primers with a specificity for exons 1 and 3 of CYP4Z1 amplified two CYP4Z1 splice variants (1 and 2) in 3/5 HL cells lines but not in normal peripheral blood mononuclear cells (PBMC). Primers with a specificity for exon 10 and the adjacent HERV-like sequence detected transcripts (3) with an alternative transcription start site only in HL cell lines but not in PBMC; ntc, no template control; M, DNA size marker. **(C)** Schematic representation of identified CYP4Z1 transcripts. Exons are indicated by blue boxes. The position of the HERV-like sequence is indicated by a red box.

We identified and sequenced three different transcripts (Figure 5) and found that the two longer transcripts represent CYP4Z1 splice variants with or without exon 2. Primers with a specificity for exons 1 and 3 of CYP4Z1 demonstrated the presence of these two CYP4Z1 splice variants in 3/5 HL cells lines but not in normal peripheral blood mononuclear cells (PBMC).

The shortest transcript corresponds to a sequence that starts in the intron between exons 9 and 10 of CYP4Z1. The intronic part of this transcript has homology to L1 transposable elements. Primers with a specificity for exon 10 and the adjacent HERV-like sequence identified transcripts with an alternative transcription start site defined by the HERV-like sequence only in HL cell lines but not in PBMC (Figure 5).

The putative function of CYP4Z1 in HL cells is unknown. CYP4Z1 is not expressed in all HL cell lines, so it seems unlikely that CYP4Z1 plays a major role in HL pathogenesis. Nevertheless, as recently discussed for breast cancer, CYP4Z1 might represent an interesting target for cancer therapy (Yang et al., 2017).

For example, the enzymatic function of CYP4Z1 might be targetable for prodrug activation. In addition, autoantibodies against CYP4Z1 have been detected in breast cancer patients, suggesting that CYP4Z1 can serve as target for immunological treatment strategies.

The relevance of the CYP4Z1 splice variant with missing exon 2 is unclear. Splice site analysis of the genomic CYP4Z1 reference sequence (NG_007967.1) using Human Splicing Finder (Desmet et al., 2009) yielded the expected splice donor site (GAGgtaaga) at the 3′ end of exon 1 with an HFS score of 95.9 and a MaxEnt score of 10.06.

Interestingly, however, the 5′ end of exon 2 yielded a splice acceptor site HSF score of 83.63 and a MaxEnt score of 5.55, whereas the 5′ end of exon 3 showed an HSF score of 89.69 and a MaxEnt score > 12. Hence, it might be possible that the exon 3 acceptor is preferentially used, and exon 2 is lost, resulting in the truncation of the protein sequence.

Alternatively, a downstream start codon might be used, resulting in an N-terminally truncated CYP4Z1 protein. The lost amino acids include the transmembrane region, and it might be possible that the new splice variant represents a soluble isoform of CYP4Z1. The presence of such soluble isoforms might be important for the development of future treatment strategies using CYP4Z1 as target.

In breast cancer, CYP4Z1 and the pseudogene CYP4Z2P have been indicated to play a role in angiogenesis and cell transformation (Zheng et al., 2015), but it needs to be analyzed if the detected HERV-associated transcript variant can interfere with the CYP4Z1/CYP4Z2P network. Interestingly, all HL cell lines that expressed the newly identified transcript variant also expressed the longer transcripts. This suggests that the expression of the new CYP4Z1 transcript variant is not controlled by the HERV-like sequence independently from the normal promoter but that the activity of the locus as a whole is switched on in CYP4Z1 expressing HL cells.

This application example demonstrates that WebHERV might be useful for the analysis of gene expression associated with HERV-like sequences. The performed analyses preferentially returned hits that have a relatively high sequence similarity with preserved HERV-like sequences, whereas shorter sequences, isolated long terminal repeats, or HERV-like sequences with unclear phylogenetic relationships to retroviruses such as members of the transposon-like human element (THE) family were not recognized entirely.

Hence, we included the RepeatMasker coordinates of LTR elements as an alternative database in the WebHERV server. Using this database allows the identification of additional HERV-like sequences including for example the mentioned THE family.

These elements can play an important role in the context of human diseases including HL. One example is a long terminal repeat of the THE1B family acting as promoter for the colony stimulating factor 1 in HL cells (Lamprecht et al., 2010). As described in the online GitHub documentation, WebHERV can be extended to include also broader arrays of elements beyond HERV-like sequences.

The feature of WebHERV to provide pre-processed HERV loci might be advantageous for some users, but other user might be interested in extending or replacing the lists of genome coordinates with further or alternative putative elements. Hence, WebHERV provides the possibility of including additional elements as well as additional genome sequences for users who wish to perform similar studies with elements of their choice in genomes of their choice.

## Author contributions

MN, MS, and IG designed the study. KK, MN, MS, and NW performed the experiments. All authors analyzed the data, wrote the manuscript, and approved the final version of the manuscript.

### Conflict of interest statement

The authors declare that the research was conducted in the absence of any commercial or financial relationships that could be construed as a potential conflict of interest.

## Nomenclature

### Resource identification initiative

Used cell lines: HDLM-2: DSMZ Cat. ACC-17, RRID:CVCL0009. KM-H2: DSMZ Cat. ACC-8, RRID:CVCL1330. L-1236: DSMZ Cat. ACC-530, RRID:CVCL2096. L-428: DSMZ Cat. ACC-197, RRID:CVCL1361. L-540: DSMZ Cat. ACC-72,RRID:CVCL1362.
