# Supplementary material for: WebHERV: A Web Server for the Computational Investigation of Gene Expression Associated With Endogenous Retrovirus-Like Sequences
Source: Front Microbiol. 2018 Nov 5;9:2384. doi: 10.3389/fmicb.2018.02384 (PMC6231192; doi:10.3389/fmicb.2018.02384)
Supplement: Supplementary file 1 [file Data_Sheet_1.PDF]

WebHERV: A web-server for the computational investigation of gene expression associated with endogenous retrovirus-like sequences

Konstantin Kruse, Martin Nettling, Nadine Wappler, Alexander Emmer, Malte Kornhuber, Martin S. Staege, Ivo Grosse

Supplementary Material

1. Supplementary Figure 1. Examples of up-regulated genes in HL cell lines in comparison to B cells. Page 2

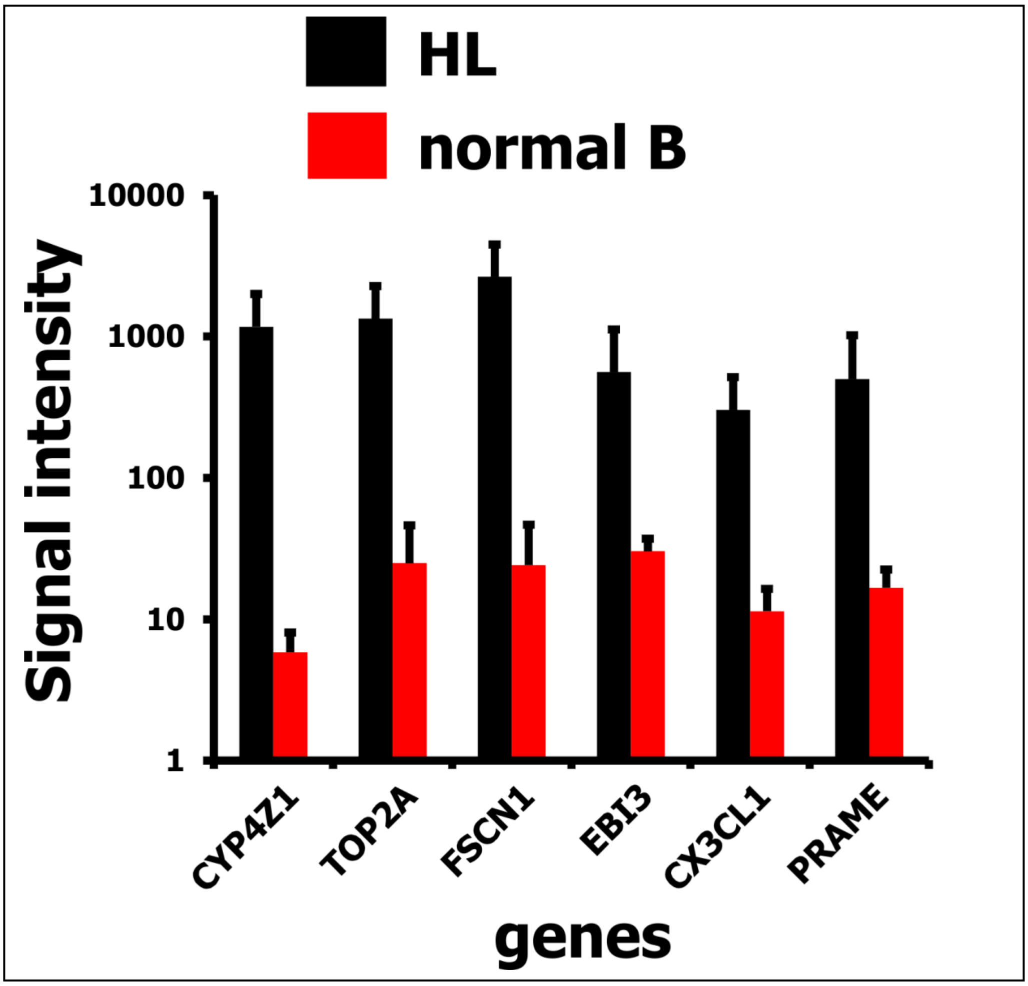

Supplementary Figure 1. Examples of up-regulated genes in HL cell lines in comparison to B cells. Presented are means and standard deviations for probe sets identified as associated with HERV-like elements in HL cell lines (closed bars) and normal B cells (red bars; GEO data set 20200).
